# A Monolithic 3D Printed Axisymmetric Co-Flow Single and Compound Emulsion Generator

**DOI:** 10.3390/mi13020188

**Published:** 2022-01-26

**Authors:** Amirreza Ghaznavi, Yang Lin, Mark Douvidzon, Adam Szmelter, Alannah Rodrigues, Malik Blackman, David Eddington, Tal Carmon, Lev Deych, Lan Yang, Jie Xu

**Affiliations:** 1 Department of Mechanical and Industrial Engineering, University of Illinois at Chicago, Chicago, IL 60607, USA; aghazn2@uic.edu; 2 Department of Mechanical, Industrial and Systems Engineering, University of Rhode Island, Kingston, RI 02881, USA; yanglin@uri.edu; 3 Physics Department and Solid-State Institute, Technion, Haifa 3200000, Israel; mark.douvidzon@technion.ac.il; 4 Department of Biomedical Engineering, University of Illinois at Chicago, Chicago, IL 60607, USA; szmelte1@uic.edu (A.S.); arodr251@uic.edu (A.R.); dte@uic.edu (D.E.); 5 Department of Mechanical Engineering, Carnegie Mellon University, Pittsburgh, PA 15213, USA; malikbla@andrew.cmu.edu; 6 School of Electrical Engineering, Tel Aviv University, Tel Aviv-Yafo 6997801, Israel; total@tauex.tau.ac.il; 7 Physics Department, Queens College of CUNY, New York, NY 11367, USA; lev.deych@qc.cuny.edu; 8 Department of Electrical and Systems Engineering, Washington University, St. Louis, MO 63130, USA; lyang25@wustl.edu

**Keywords:** emulsion generator, compound droplets, 3D printing, microfluidic device, microfluidics

## Abstract

We report a microfluidic droplet generator which can produce single and compound droplets using a 3D axisymmetric co-flow structure. The design considered for the fabrication of the device integrated a user-friendly and cost-effective 3D printing process. To verify the performance of the device, single and compound emulsions of deionized water and mineral oil were generated and their features such as size, generation frequency, and emulsion structures were successfully characterized. In addition, the generation of bio emulsions such as alginate and collagen aqueous droplets in mineral oil was demonstrated in this study. Overall, the monolithic 3D printed axisymmetric droplet generator could offer any user an accessible and easy-to-utilize device for the generation of single and compound emulsions.

## 1. Introduction

In recent years, emulsions generated in microfluidics have been exploited extensively in different areas including single-cell analysis [1,2], drug discovery [3,4], therapeutic agent delivery [5], medical diagnostics [6,7], the food industry [8], and nano- and microscale particle synthesis [9,10,11,12]. In addition, emulsions can play a significant role in the emerging field of opto-microfluidics. The softness of the droplet surfaces offers a unique structure for trapping light into a so-called Whispering Gallery Mode (WGM) [13,14]. In such a mode, the droplets serve as soft resonators, exhibiting unique optomechanical properties [15]. While many general applications of droplets require droplets within the size of 100 μm [16,17], in special applications, such as high-energy-density physics [18], or the fabrication of inertial confinement fusion (ICF) targets [19], the need for the production of thin-walled droplets with diameters measured in millimeters is playing a significant role. Additionally, in biomedical applications, millimeter-scale hydrogel droplets have the potential to increase reproducibility in cancer organoid culturing and passaging [20]. Therefore, the generation of single or compound droplets with millimeter-scale diameters is important in various applications.

The generation of emulsions is essentially a process in which two or more immiscible fluids are forced to mix with each other. The wide applications of emulsions call for a convenient method to produce the emulsions on or off the chips. Various configurations such as cross-flow, flow-focusing, and co-flow structures have been demonstrated and different fabrication techniques have been used to produce passive chips in which the interface of the emulsion is deformed under certain flow fields, and the pinch-off process of the emulsion is naturally achieved as a result of interfacial instabilities. In active chips, local actuations, such as thermal, electrical, magnetic, acoustic, and mechanical forces, can be exerted during the breakup process of emulsion for the microfluidic emulsification [21,22]. The single and compound emulsion generation processes are significantly dependent on the fluid’s properties (viscosity, density, etc.) and the hydrodynamic forces applied through the interaction of the fluids. Numerous studies have been conducted on the mechanism of droplet generation in microfluidic devices. Some authors classified the formation mechanism into the confined, unconfined, and partly confined breakup mechanisms, which are called the squeezing, dripping, and transition regimes, respectively [23,24,25]. The droplet generation process is dominated by interfacial tension and can be divided into growing and detaching stages. These stages stem from the competition of interfacial tension and viscous shear. The detachment of the droplet from the orifice occurs when the droplet volume reaches the critical point such that the interfacial tension cannot hold the semi-spherical interface of the droplet attached to the orifice against the viscous shear of this continuous phase. Thus, the pressure inside the interface builds up along with necking of the droplet and drives the droplet towards the high-pressure regime. This would result in the detachment of the droplet from the neck [26,27]. After the droplets detach, the viscous shearing force due to the continuous phase carries the droplets downstream.

The process of fabricating a microfluidic device requires the application-driven selection of materials in coordination with appropriate fabrication methods. Currently, there are many techniques for the fabrication of microfluidic chips, such as polymer-based replica molding, glass/silicon-based micromachining, commercial modular assembly, and newly developed 3D printing. There are pros and cons in using each aforementioned fabrication techniques in microfluidic chip production. The micromachining process on glass/silicon-based materials offers thermal stability under high temperatures for droplet-based chemical syntheses, and it is solvent-resistant in an organic environment for many droplet-based reactions. In addition, the specific properties of this fabrication method on glass/silicon-based materials offer high thermal conductivity, and electroosmotic flow stability [21]. On the other hand, the large-scale production of microfluidic chips using this fabrication technique is hindered by its high cost, the demand for cleanroom equipment, and its time-consuming process. Using polydimethylsiloxane (PDMS) in molding replication offers intrinsic advantages such as a simple sealing process using air or oxygen-plasma oxidation without implementing either high temperatures or pressure [28]. However, PDMS has its limitations in some aspects; for instance, its small molecular adsorption, high-pressure intolerance, and organic incompatibility hinders the usage of this material in certain situations. The commercial modular assembly technique offers the flexibility of using different devices, such as commercial connectors, glass capillaries, and needles, to build a droplet microfluidic chip [29,30]; however, assembling such items requires manual effort and preparation, which is prone to error. Another technique which has been flourishing in the field of microfluidics is 3D printing. Researchers have increasingly begun using this technique as a promising way to fabricate microfluidic devices because it offers the freedom to design novel structures, the capability of building truly 3D structures, the possibility to easily implement design ideas using digital design tools, and it provides fast prototyping [31].

Numerous advantageous of the use of 3D printing technology in the field of microfluidics have provided the freedom of design and the usage of different raw materials and printers for the researchers to produce a variety of devices. In particular, it has been used for making devices to produce emulsions, and the empirical knowledge of the droplet formation mechanisms have been discussed in these studies; however, knowledge is still lacking in the design and optimization of the droplet formation process to fully harness the advantages of 3D printing [32]. Specifically, single chips and existing structures of droplet microfluidics have been reproduced using different 3D printing raw materials. Hybrid and modular devices were created using combined tubing or glass capillaries, and smaller droplets could be produced. Integrated devices were used to facilitate the fabrication process, which was difficult using conventional methods. High-throughput droplet generation was reported to demonstrate parallel droplet generation in droplet microfluidic devices. Three-dimensional printed fittings and modules were designed and fabricated to produce single and compound emulsions using needles [33]; a modular device was produced using different printers, and compound emulsion generation was produced [34]. Moreover, a non-planar emulsion generator using T-junction structures was demonstrated using stereolithography (SLA) 3D printers [35,36]. In another study, fused filament fabrication was used to produce a droplet microfluidic device [37]. One major drawback of all these mentioned designs is the complexity of the assembly processes.

To unleash the full potential of 3D printing technology in droplet microfluidics in terms of extreme simplicity, a one-piece device with a co-flow design of channels for generating single and compound emulsions has been produced in the present study. This device has been designed in a monolithic manner and the fabrication process has been performed using a commercial affordable SLA 3D printer. The production of various sizes of single emulsions and the performance of the emulsion generation have been studied. The device has also been demonstrated to produce compound emulsions, with a different number of encapsulated emulsions using the passive method and by controlling the flow rates of three different phases. We believe the simplicity of the design, fabrication, and manipulation of our device would help researchers to achieve complex droplets for various applications.

## 2. Materials and Methods

The designed device is printed with an inexpensive commercial 3D printer, Form3 (Formlabs, Inc., Somerville, MA, USA), which only costs around $3000, and it is based on the stereolithography (SLA) technique to print structures. SLA printers use light-reactive thermoset materials called “resins” which form short molecular chains when they are exposed to certain wavelengths of light. Rigid or flexible geometries would be formed by joined molecular chains, polymerized monomers, and oligomers. This printing method is by far the most popular technique for 3D printing in the microfluidics field of study since it is cheap and easy to use; it also has the capability of printing overhanging structures, such as channels, providing a smooth surface and relatively good resolution in comparison with other printing techniques [38]. More importantly, the great advantage of using an SLA printer is the possibility of working with a clear resin that provides excellent transparency, allowing researchers to observe the motion of fluids inside the printed channels. For tuning and optimizing the encapsulation of inner emulsions, we need visual control that can be achieved by using transparent materials such as PDMS and clear resin. Therefore, we chose to use clear resin in the Stereolithography 3D printer to observe and achieve appropriate flow rates for the generation of emulsions. In addition, the Young’s Modulus of post-cured clear resin is 2.8 GPa, which is much greater than that of PDMS (0.75 × 10^−3^ GPa). Therefore, the device is immune to deformation, especially at high liquid flow rates and pressures, minimizing performance fluctuation. To achieve the best resolution and transparency using the printed device, we chose the highest possible resolution of the 3D printer, which is 25 microns. This resolution defines each layer’s thickness in the process of printing, and the device would be perfect for microfluidic droplet generation, providing results with a smooth surface finish and transparent microfluidic droplets.

In comparison, the conventional 2D planar droplet generators face a major challenge in making double emulsions due to the need to alter the wettability of the channels at each stage of the two-stage droplet generation process. As a result, achieving the integration of both hydrophobic and hydrophilic surfaces may entail a complex assembly of multi-materials, or functionalizing the surface of channels. Thus, by implementing the 3D printing technology in the production of our device, we could generate a truly 3D device where the inner fluid is always kept in the middle, preventing the wetting on any unfavorable surfaces during the emulsion generation process.

**Figure 1:**
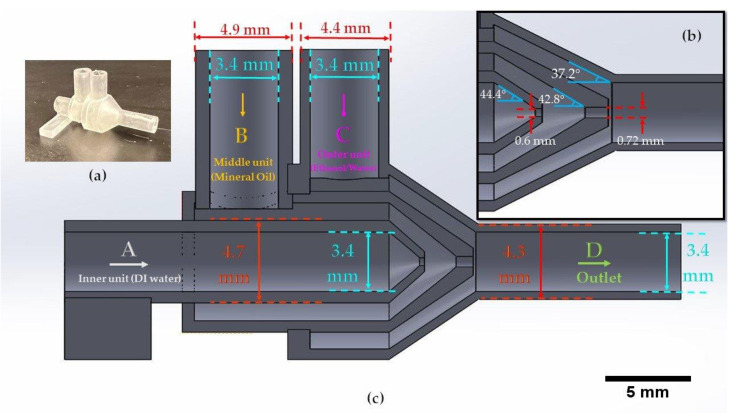
(**a**) The actual 3D printed device. (**b**) The dimensions and angles of the orifice for units A and B. (**c**) The dimension information on the 3D printed device units, including unit A: inner unit (DI water), unit B: middle unit (oil), and unit C: outer unit (ethanol-water) of the monolithic 3D printed device.

The design of the channels is based on preventing the clogging of uncured resin in the device. The channels in each unit are designed with a 1 mm dimension to ensure enough space for the resin to flow out of the device while printing. Also, the printing orientation of the device was considered as vertical to overcome the clogging issue. For the design of the orifices, we could successfully fabricate 600 and 720 μm orifices without clogging; however, we tried smaller dimensions for the inner orifice, and the success rate was lower since printing smaller channels is highly dependent on the specs of the printer. Therefore, for a higher printing success rate of the inner orifice, we kept the inner orifice size at 600 μm for the proof-of-concept purpose in this study. The dimensions of the inlets and outlets were designed according to the diameters of the tubes we used. The inlets were designed with an inner diameter (ID) of 3.4 mm for the tight connection of the tubes and the outlet has a 4.7 mm outer diameter (OD) for the tight connection of the ID of the soft clear tube. We chose a bigger tube for the outlet to prevent the contact of droplets with the tube walls. One factor to consider in designing a microfluidic device for STL 3D printing is the clogging issue of the channels during the curing of the resin. In the 3D printing process, the resin is cured using an ultraviolet (UV) light, with 250 mW power and 405 nm wavelength in a compact system of lenses and mirrors to deliver accurate prints. The channels should be designed so that resin can flow out during printing; otherwise, it can clog in the channel, which is not desirable. To minimize clogging, we printed the device vertically to let resin flow out of the orifice since resin, which is a liquid, tends to drip. In addition, to prevent the clogging of the channels, we designed the smallest channel of the device, Unit A’s orifice (Figure 1) with a 600 μm diameter, as small as possible for the printer to print without clogging. Washing the final product with isopropyl alcohol (IPA) dissolves uncured resin and prevents blockage of the channels in the device.

In our study, we are focused on the generation of emulsions using the 3D printed device and the demonstration of the device’s ability to produce various emulsions. Figure 1 is demonstrating the dimension of the units inside the device and the different units for the infusion phases. We first showed the generation of a single emulsion and the different sizes of emulsions which we could achieve by using the 720 μm orifice size in different flow rates. Then, we demonstrated the production of double and compound emulsions in different flow rates and various numbers of water emulsions encapsulated in oil emulsions. For the stable production of the emulsions, a vertical holder was used to grab the device and hold it vertically during the entire experiment. Firstly, the device was primed with the continuous phase (using a water-ethanol solution) to remove air. Then the dispersed phase (oil) was introduced. Since the continuous phase first wets all the areas of unit B and the orifice, the dispersed phase reaches downstream without touching the walls, thanks to the 3D axisymmetric design. A similar process is used to set up the device for double emulsion generation.

Using the printed device, we successfully generated single, double, and compound emulsions of water and oil. We used three different liquids, namely, deionized water, mineral oil (UltraPro 01651), and ethanol, for generating different combinations of emulsions including single oil-in-water (O/W) emulsions, double, and compound water-in-oil-in-water (W/O/W) emulsions. During this process, we added surfactants to the continuous phases for stabilizing the emulsions and preventing the coalescence and rupture of the emulsions. To investigate and measure the size of the emulsions, we collected them in a glass beaker that was filled with the continuous phase fluid. In the production of single and double emulsions, using appropriate surfactants plays an important role in the stability of the emulsions. We were able to stabilize the O/W emulsions by using polyglycerol polyricinoleate 90 (PGPR 90) 1% (Danisco, Copenhagen, Denmark) in mineral oil and by using a solution of ethanol-water with 0.1% of sodium dodecyl sulfate (SDS) (Fisher BioReagents, Waltham, MA, USA). In addition, for producing double/compound emulsions, we used deionized water as the most inner phase to generate W/O/W emulsions. The liquids were infused to the device by using three syringe pumps (Chemyx 3000 Nexus, Stafford, TX, USA), commercial syringes (Becton, Franklin Lakes, NJ, USA), and clear Tygon tubes (Saint-Gobain, Courbois, France) for directing the liquids to the device. Collected emulsions were observed via a Nikon Eclipse Ti-S microscope and pictures were taken by a Phantom Miro M310 camera which was mounted on the microscope. The evaluation of the size of the emulsions was performed using ImageJ software.

## 3. Results and Discussion

### 3.1. Single Emulsion Generation

The first step we took for the investigation of producing different sizes of emulsions was to analyze the single emulsion’s behavior during the production process, which is the requirement for achieving satisfactory results for the next step, the double and compound emulsions generation. To investigate the impacts of varying the dispersed and continuous phase flow rates on the droplet size, we considered three different flow rates for the dispersed phase, and we kept increasing the continuous flow rate for each of these flow rates. After each set of flow rates, we collected the emulsions and took their pictures using a microscope with integrated camera. Then, we measured the size of droplets with ImageJ (National Institute of Health, Bethesda, MD, USA) software.

**Figure 2:**
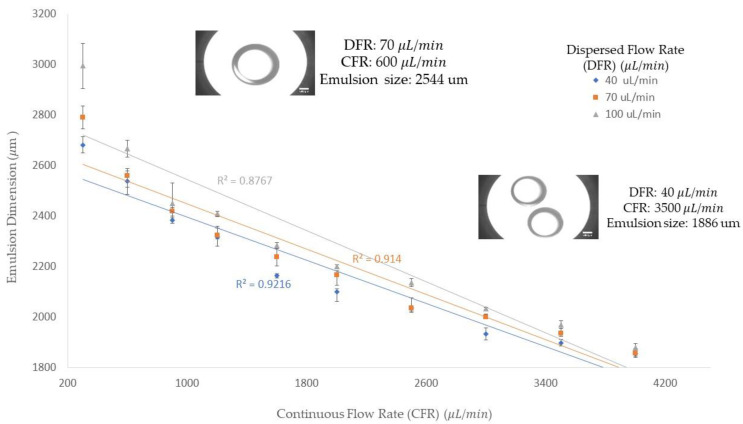
Emulsions generated in three different dispersed flow rates (40, 70, and 100 μL/min) and various continuous flow rates (from 300 μL/min to 4000 μL/min). The *y*-axis is the emulsion size generated in micrometers and the *x*-axis is the continuous flow rate in microliters per minute (μL/min). The dispersed flow rates in this figure are shown in blue diamonds (40 μL/min), orange squares (70 μL/min), and grey triangles (100 μL/min).

Figure 2 shows the diameter of the collected emulsions corresponding to the dispersed and continuous flow rates. In the experiment, we were able to achieve an emulsion size from 1838 to 3090 μm by varying the dispersed and continuous flow rates. From Figure 2, we can see that when the dispersed phase flow rate is fixed, the droplet size decreases with increasing continuous flow rates. In addition, the trend line shows a decline trend that indicates the droplet size decreases by increasing the continuous flow rate. The impact of changing the dispersed flow rate and keeping the continuous flow rate constant is seen in Figure 2. From this figure, we can conclude that the higher dispersed flow rate at a constant continuous flow rate would result in a bigger size of the emulsions. This fact is due to the increase in the amount of emulsion and as a result, it requires more viscous force to detach the emulsion. Since, the viscous force is constant, due to a constant continuous flow rate, the emulsion grows bigger and finally pinches off, turning into a stable form. All three trendlines, which correspond to three different dispersed flow rates, follow a declining trend. Therefore, we can conclude that in the dripping regime, by increasing the continuous flow rate, we can achieve a smaller emulsion size. In addition, the emulsion size difference among the three dispersed flow rates becomes smaller when the continuous flow rate value increases. Therefore, in higher continuous flow rates, the emulsion size would be more uniform due to the significance of the drag force in the mechanism of emulsion generation.

**Figure 3:**
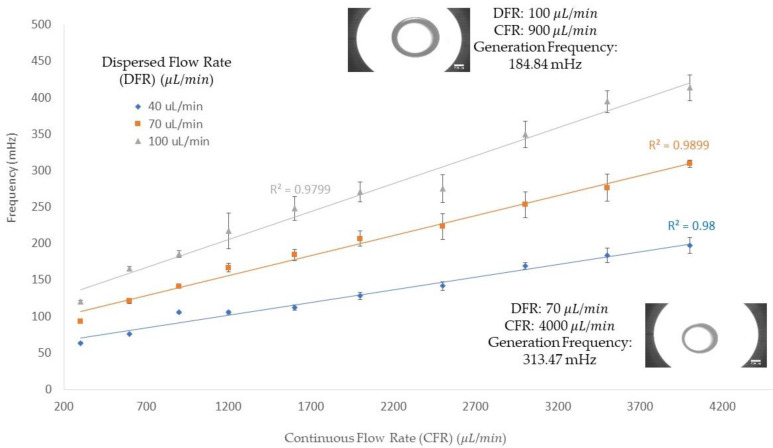
The continuous flow rate (μL/min) and frequency (mHz) of single emulsion generation at three different dispersed flow rates (40, 70, and 100 μL/min) and various continuous flow rates (from 300 μL/min to 4000 μL/min). The *y*-axis is the frequency of single emulsion generation in millihertz, and the *x*-axis is the continuous flow rate in microliters per minute (μL/min). The dispersed flow rates in this figure are shown in blue diamonds (40 μL/min), orange squares (70 μL/min), and grey triangles (100 μL/min).

In Figure 3, the frequency of the single emulsion generation in three different dispersed flow rates (40, 70, and 100 μL/min) is demonstrated on the *y*-axis along with the *x*-axis for continuous flow rates ranging from 300 to 4000 μL/min. The frequency of the emulsion generation increases with increasing the continuous flow rate when the dispersed phase flow rate is kept constant. For instance, with a 40 μL/min dispersed flow rate and a 300 μL/min continuous flow rate, the frequency of the generation obtained was 63.62 mHz; by increasing the continuous flow rate to 4000 μL/min, the frequency of the dispersed flow rate was increased to 197.26 mHz. In addition, the impact of increasing dispersed flow rates on the frequency of single emulsion generation in a constant continuous flow rate is shown in this figure: in a 300 μL/min continuous flow rate, with dispersed flow rates of 40, 70, and 100 μL/min, the frequency was obtained at 63.62, 96.06 and 119.96 mHz, respectively. In another continuous flow rate, 4000 μL/min, frequencies for dispersed flow rates of 40, 70, and 100 μL/min were 197.26, 309.33, and 413.71 mHz, respectively. Therefore, the increase in either the dispersed or continuous flow rate resulted in a higher frequency production of single emulsion, as shown in Figure 3. These results were obtained using a 720 μm orifice and the flow rates were chosen based on significant changes in the diameter of the emulsions generated during the experimentation.

### 3.2. Double and Compound Emulsion Generation

We extended our experiments to investigate the performance of the 3D printed device by infusing the inner phase (deionized water) to produce double emulsions (mono encapsulation of emulsions), and compound emulsions (poly encapsulation of emulsions). The compound emulsions are composed of a primary large emulsion which includes a single or multiple smaller emulsions. These small emulsions are kept isolated from the surroundings, wrapped in the outer immiscible fluid. In our experimentation, the inner liquid was infused to the unit A with a lower flow rate than the middle phase, since the middle phase should play the continuous phase role for the dispersed deionized water. Therefore, the infusion of three liquids, deionized water as the inner phase, mineral oil as the middle phase, and the water-ethanol solution as the outer phase, took place in our experiments by considering the lowest flow rate for the inner phase and higher flow rates for the middle and outer phases. In our microfluidic system, we could observe the dripping regime, which is characterized by the formation of droplets not far away from the orifice. Double and compound emulsion drops would detach from the inner orifice when the detaching forces of the middle phase exceeded the holding force, and the inner droplets would accumulate inside the middle phase droplet until it reached a volume where interfacial tension could not hold the droplet to the outer orifice.

**Figure 4:**
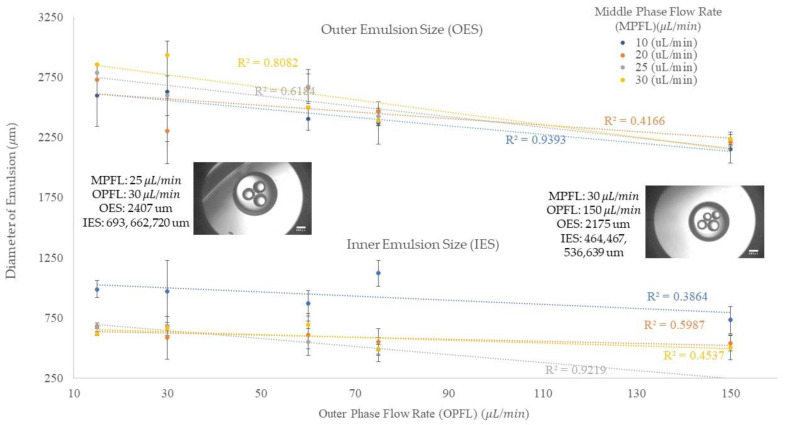
The inner and outer emulsions sizes at 2 μL/min inner the phase flow rate in different middle and outer flow rates. The outer emulsion size is shown in the upper section and the inner emulsions size is shown in the lower section of this figure.

The experiment started with the middle phase flow rate of 10 μL/min and the outer flow rate of 30 μL/min. To produce inner emulsions in a dripping regime, we chose 2 μL/min (Figure 4) as the flow rate of the deionized water since it has lower value than the middle phase. In the process of compound emulsion generation, the water-in-oil (W/O) emulsion was generated at the first orifice, unit A’s orifice, and then it moved to the second orifice, unit B’s orifice, where the oil-in-water (O/W) emulsion was generated. Since the orifice of unit A is smaller than that of unit B, the emulsion size of W/O can be smaller, and it can be encapsulated by the O/W emulsion. In addition, by tuning the middle phase flow rate, the smaller size and a greater number of W/O emulsions can be encapsulated. This is discussed in detail in the following sections.

### 3.3. Evaluation of the Double and Compound Emulsions Sizes

The double/compound emulsion generation process is more complex than the single emulsion generation since one more phase is added to the process. The generated O/W emulsions can include single or multi water emulsions to provide a water-in-oil-in-water (W/O/W) emulsion. As a result, the number and size of the inner and outer emulsions are dependent on different factors.

In the process of double/compound emulsion generation, the size of the inner and outer emulsions and the number of the inner emulsions are highly dependent on the flow rates since the drag force of the continuous phase is dominating the interfacial force and causing the detachment of the emulsion from the orifice, and the increase in the flow rate of the continuous phase is playing a significant role in this process. In addition, the increase in the dispersed flow rate can also result in the change of the size and number of droplets. Figure 4 shows the outer and inner sizes of the emulsions in different middle and outer flow rates. The results are shown separately in this figure by the upper and lower data sets. The upper part indicates the outer emulsion size and the lower part shows the inner emulsion size. This figure shows that the inner flow rate was kept constant, 2 μL/min, to investigate the impact of the increase in middle and outer flow rates on the emulsion size. The vertical axis shows the emulsion size and the horizontal axis shows the outer flow rate. In our experiment, we started with a middle phase flow rate of 10 μL/min and an outer phase flow rate of 30 μL/min. Then, the middle phase and outer phase flow rates were increased from 10 μL/min to 20, 25, and 30 μL/min, and 30 μL/min to 60, 75, and 150 μL/min, correspondingly. In the legend, the middle phase flow rates are shown in different colors.

The outer emulsion size, O/W, can be impacted by different factors in the production process. The upper part of Figure 4 indicates the behavior of the outer emulsion in various middle and outer phases flow rates. We can understand from this figure that by increasing the outer flow rate, the outer emulsion size becomes smaller. In addition, when we compare two emulsions in a same outer flow rate situation, the bigger emulsion size, O/W, could result from three possible situations. These situations can happen at a same time, or one of them can result in the bigger size of O/W emulsion. These situations can be classified as (1) the emulsion encapsulated a higher number of inner emulsions, (2) the average inner emulsion size was larger, and (3) the flow rate of the middle phase was higher.

At the outer flow rate of 15 μL/min, the increase in the middle phase flow rate resulted in a higher number of inner emulsions and a larger outer emulsion size. When we increased the outer flow rate to 30 μL/min, the 30 μL/min middle phase flow rate still showed a larger outer emulsion size. In addition, the middle flow rate of 25 μL/min shows a higher flow rate and number of emulsions, along with larger inner emulsions than the 20 μL/min flow rate, so it shows a larger outer emulsion size. The 10 μL/min shows an average size close to 25 μL/min and larger than 20 μL/min because of its significantly larger inner emulsion and slower flow rate, both of which result in the bulging of the outer emulsion. In addition, the significant difference in both inner and outer emulsion size at 20 μL/min is due to small inner emulsion which was resulted by the incomplete pinch off process. It means that during this process the second inner emulsion was broken into two parts and one part encapsulated in the oil emulsion and the other part encapsulated in the next emulsion.

In three other outer flow rates, 60 μL/min, 75 μL/min, and 150 μL/min, the 10 μL/min middle flow rate shows a smaller outer emulsion since it has a larger inner emulsion, and the oil emulsion pinches off faster, as soon as the inner emulsion enters it. At the outer flow rate of 60 μL/min, the 30 μL/min middle flow rate shows a smaller outer droplet size since it includes a smaller number and a bigger size of droplets, the same as the 10 μL/min middle flow rate as described. For the 20 μL/min and the 25 μL/min middle flow rates, these showed a similar average size on the outer emulsion, but a difference in the number and size of the emulsions included so that the 25 μL/min middle flow rate shows smaller and more inner emulsions. After increasing the outer flow rate to 75 μL/min, the 30 μL/min and 25 μL/min middle flow rates show similar behavior in the inner and outer average emulsion sizes and the number of inner emulsions. This is due to the regime that the inner phase enters that requires higher/lower drag force for making significant changes in the size and number of emulsions. The 20 μL/min middle flow rate in this situation shows less emulsions, but a larger average size, resulting in faster pinch off since the oil drop reaches to the point in which the drag and inertia forces become significant in helping the process. For the last scenario, all the middle phase flow rates show the same results for the 150 μL/min outer flow rate as they did in the 15 μL/min outer flow rate.

**Figure 5:**
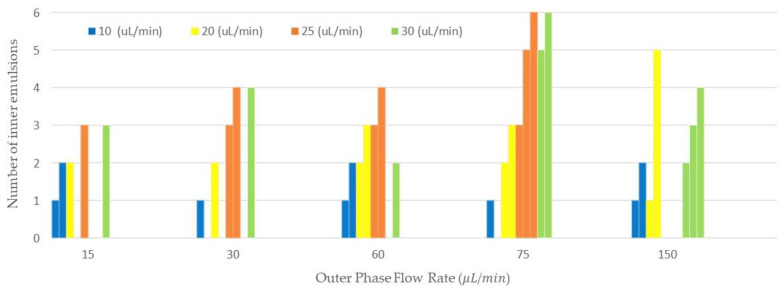
The number of inner emulsions in the double/compound emulsion generated with the 3D printed device.

The generation of the inner emulsion in compound emulsions is same as the generation of a single emulsion since the inner flow rate is considered as a dispersed flow rate for the continuous middle phase flow rate. Therefore, an increase in the inner phase flow rate at a constant continuous middle phase flow rate would result in an increase in the emulsion size and the frequency of generation, as discussed in the “Single Emulsion Generation” section. In our experiments, we kept the inner flow rate at a low constant flow rate (2 μL/min) and increased the middle and outer flow rates to investigate the encapsulation of water emulsions in oil emulsions. By increasing the middle phase flow rate from 10 to 30 μL/min, we could not observe significant changes in the inner emulsion size because of the geometrical limits of device (the orifice size, the distance between two orifices, and the angle of the interior units). Therefore, we decided to continue the experimentation with these four sets of middle phase flow rates and we kept the inner flow rate constant, since increasing the inner phase flow rate results in a higher frequency of generation and a larger size of emulsions. As we discussed in the “Single Emulsion Generation” section, at a constant dispersed flow rate, the increase in the continuous flow rate would result in smaller emulsions; this fact was expected in the generation of the inner emulsions, as well as their encapsulation in the oil emulsions. However, we speculated that there would be exceptions, such as those shown at the outer flow rate of 30 μL/min, and the middle phase flow rate of 20 μL/min when a smaller average droplet size was shown. This is due to the partial encapsulation of the inner emulsions at 20 μL/min in the middle phase flow rate and resulting in the smaller average size of emulsions. In another case, at the 30 μL/min middle phase flow rate, two uniform inner emulsions larger than those at the 20 μL/min and 25 μL/min middle flow rates were generated at the 2 μL/min inner flow rate, 30 μL/min middle flow rate, and 60 μL/min outer flow rate, based on our observations. This data is also shown in Figure 5 with a green bar.

### 3.4. Evaluation of the Number of Emulsions Generated

We collected double and compound emulsions in different sets of flow rates and demonstrated the number of encapsulated W/O emulsions in an O/W emulsion as shown in Figure 5. The vertical axis corresponds to the number of inner emulsions and the horizontal axis shows the outer flow rate. The middle phase flow rates are shown in the legend of this figure. The variation of the number of encapsulated emulsions is tunable, to some extent, by the tuning of the flow rates. In addition, we showed examples of compound emulsions collected with different number of water emulsions encapsulated in mineral oil in Table 1. Middle and outer flow rates, inner and outer emulsion size, and average and standard deviation of the inner emulsions are shown in this table.

In Figure 5, the number of inner emulsions in the double/compound emulsions generated via the 3D printed device is shown. Here, we tried to investigate the effect of the flow rate on the number of emulsions encapsulated. In general, the increase in the continuous flow rate would result in a higher frequency of emulsions generation, since the generated inner emulsions would be accumulated in the oil (outer emulsion) until they pinched off from the orifice and produced double/compound emulsions. In addition, the increase in frequency of the inner emulsion production would result in more inner emulsion encapsulation in the outer emulsion. As we can see in Figure 5, the number of inner emulsions was increased by increasing the middle phase flow rates. For instance, the increase in the middle phase flow rates of 25 μL/min and 30 μL/min at the outer flow rate of 15 μL/min resulted in the generation of three inner emulsions. In some cases, we could observe various numbers of inner emulsions because of the encapsulation process. This process can behave differently in some situations due to untuned flow rates and different inner emulsions sizes. For instance, in a set of flow rates with a middle flow rate of 25 μL/min and outer flow rate of 150 μL/min, we could not observe any compound emulsion encapsulation since encapsulation of inner emulsions was not tuned for this set of flow rates.

### 3.5. Frequency of the Emulsion Generation

**Figure 6:**
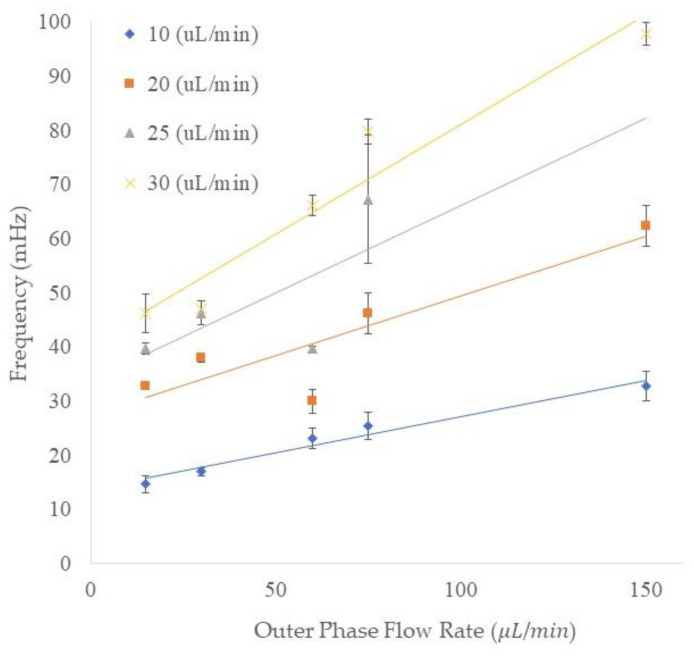
The frequency (mHz) of the double/compound emulsions generation in different middle and outer flow rates (μL/min). The *y*-axis shows the frequency of emulsion generation in millihertz, and the *x*-axis shows the outer phase flow rates. The legend of this figure indicates the middle phase flow rate.

The frequency of the outer emulsion generation is shown in Figure 6. The vertical axis shows the frequency of the generated double emulsions and the horizontal axis indicates the outer flow rate. The data points shown as diamond, square, triangle, and cross symbols represent the four different middle phase flow rates. The trend of the frequency of the generation of double/compound emulsions is similar to that for the single emulsion generation since the increase in either outer or middle flow rates resulted in higher frequency. For instance, by increasing the outer flow rate from 15 to 150 μL/min, the frequency of compound emulsion production increased from 14.64 to 32.74 mHz in the 10 μL/min middle phase flow rate. In another set of flow rates (the middle phase flow rate of 20 μL/min), increasing the outer flow rate from 15 to 150 μL/min resulted in an increase of frequency from 32.8 to 62.4 mHz. In addition, we could speculate an increase in compound emulsion production frequency by keeping the outer phase flow rate constant and increasing the middle phase flow rate from 10 to 30 μL/min. For instance, at 75 μL/min outer phase flow rate, increasing the middle phase flow rate from 10 to 30 μL/min resulted in frequencies of 25.4, 46.1, 67.2, and 79.8 mHz for 10, 20, 25, and 30 μL/min middle phase flow rates, respectively. The frequency in some combinations of middle and outer flow rates can exhibit errors due to the process of encapsulation. For instance, at 75 μL/min outer flow rate and 25 μL/min middle flow rate, we can see higher fluctuation in the double emulsions generation. This is because of an untuned set of flow rates which resulted in various numbers of encapsulated inner emulsions.

### 3.6. Generation of Bio-Emulsions

Droplet microfluidics has been a powerful tool used in various investigations including single-cell studies in which single-cell assays can be used to produce monoclonal antibodies from individual hybridoma cell clones; the analysis of protein crystal growth and enzyme kinetics; as well as studies of directed evolution by in vitro selection; small-molecule synthesis in microdroplets; and drug discovery [39,40]. Additionally, in-droplet IFN-γ capture assays have recently been studied using droplet encapsulation that could help prevent the diffusion of secreted products to neighboring cells, dramatically reducing both false positives and false negatives relative to assays performed without droplets [14]. Millimeter-scale hydrogel droplets have the potential to increase reproducibility in the culture and passaging of cancer organoids. Currently, researchers growing organoids from pancreatic cancer biopsies manually resuspend tissue fragments in a basement membrane matrix and pipette them into 10 uL (2.5 mm) droplets [41]. This manual method is labor intensive, invites the opportunity for heterogeneity, and produces only a ~50% yield. Our droplet generator has the potential to automate and improve protocols such as these that require larger tissue fragments.

Alginate hydrogels have been attractive in different applications such as drug delivery, wound healing, and tissue engineering, as these gels retain structural similarity to the extracellular matrices in tissues [42]. Another famous biomaterial which has been used for many years to study drug delivery systems is collagen. Collagen is one of the most useful biomaterials. Collagen is a primary resource in medical applications thanks to its excellent biocompatibility and safety due to its biological characteristics, such as biodegradability and weak antigenicity. Collagen is utilized in a variety of ways, including in shields used in drug delivery systems in ophthalmology, in sponges for burns/wounds, in mini-pellets and tablets for protein delivery, in gel formulation in combination with liposomes for sustained drug delivery, as a controlling material for transdermal delivery, and as nanoparticles for gene delivery and basic matrices for cell culture systems [43]. By using the device presented, we could successfully generate alginate and collagen emulsions. We used 2% of alginate, fine marine colloids (FMC), and 6 milligrams per milliliter of collagen, rat tail type 1 (Advanced BioMatrix), in deionized water to generate alginate and collagen emulsions in mineral oil. To stabilize the emulsions, Span80 with 4% concentration was used in mineral oil.

**Figure 7:**
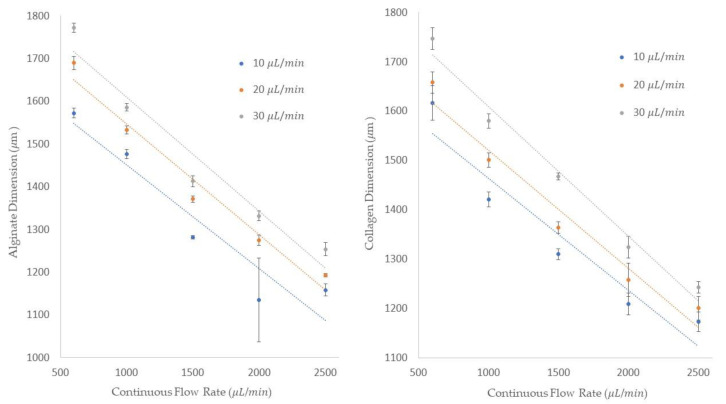
The dimensions of single emulsions for the generation of alginate and collagen emulsions.

The single emulsion generation process for both alginate and collagen were conducted under similar dispersed and continuous flow rates. We obtained the diameter of the emulsions and the frequency of the emulsion generations in our experimentation. As it is shown in Figure 7, the diameter of the alginate and collagen emulsions is shown on the *y*-axis and five different continuous flow rates are shown on the *x*-axis. Plus, we used three different dispersed flow rates, 10, 20, and 30 μL/min, as shown in the legend of these figures. Similar to the process of single emulsion generation discussed in the “Single Emulsion Generation” section, at a constant dispersed flow rate of 10 μL/min, we could generate emulsions with an average diameter of 1572.3 ± 11.7 μm at the continuous flow rate of 600 μL/min. By increasing the dispersed flow rates to 20 and 30 μL/min and keeping the continuous flow rate constant, the average emulsion size of 1689.6 ± 15.4 and 1772 ± 10.8 μm was obtained, respectively. To understand the impact of an increase in the dispersed flow rate at a constant continuous flow rate, we could attain the average diameter of 1193 ± 4 and 1253.5 ± 15.2 μm for the 20 and 30 μL/min dispersed flow rates at a 2500 μL/min continuous flow rate. Figure 7 also shows the collagen emulsion diameter using the same dispersed and continuous flow rates used in the previous experiments. For example, at a 600 μL/min dispersed flow rate, the average collagen emulsion sizes of 1617 ± 35, 1668 ± 21.1, 1723 ± 22.3 μm were obtained in 10, 20, and 30 μL/min continuous flow rates, respectively. By increasing the continuous flow rate to 2500 μL/min for all three dispersed flow rates at 10, 20, and 30 μL/min, the average diameters of 1173 ± 20, 1201 ± 23.86, and 1243 ± 12 μm were achieved. It can be concluded that by increasing the dispersed flow rate at a constant continuous flow rate, the diameter of the emulsions is increased, and if the continuous flow rate is increased at a constant dispersed flow rate, the diameter of the emulsions is decreased.

**Figure 8:**
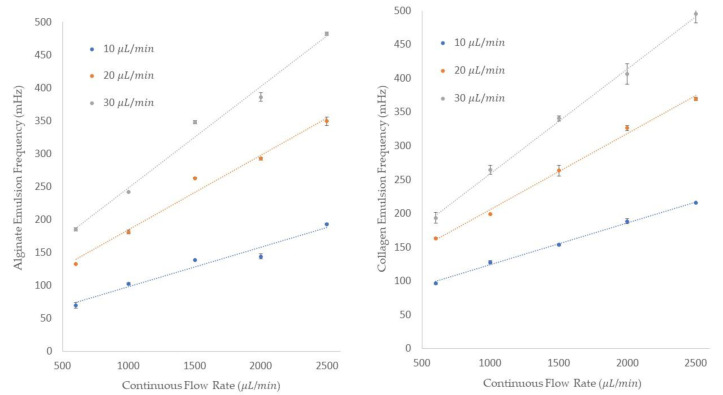
The frequency of alginate and collagen emulsions generations in mineral oil using three different dispersed flow rates with five continuous flow rates.

The frequency of single emulsions generation for both alginate and collagen emulsions is shown in Figure 8. The frequency of generation is shown on the *y*-axis and the five different continuous flow rates are shown on the *x*-axis of these figures. Additionally, the legend represents the dispersed flow rates. We could generate alginates with 69.4 ± 4.1, 132.68 ± 0.3, and 185 ± 2.6 mHz frequencies at 10, 20, and 30 μL/min dispersed flow rates and 600 μL/min of mineral oil flow rate. By increasing the mineral oil flow rate to 2500 μL/min, the frequency of the emulsion generations increased to 193.1 ± 1.2, 349.3 ± 6.4, and 482.32 ± 2.2 mHz for the dispersed flow rates of 10, 20, and 30 μL/min, respectively. For collagen generation, Figure 8 shows that at a constant continuous flow rate of 600 μL/min and three different collagen flow rates of 10, 20, and 30 μL/min, the frequencies were 96.24 ± 1.3, 163 ± 1.2, and 193.3 ± 7.8 mHz. By increasing the continuous flow rate to 2500 μL/min, the frequency of 215.5 ± 1 mHz for a dispersed flow rate of 10 μL/min, 369.45 ± 2.8 mHz for a dispersed flow rate of 20 μL/min, and 495 ± 13 mHz for a dispersed flow rate of 30 μL/min was obtained. We can see from the data provided that by increasing either the dispersed or the continuous flow rate, the frequency of the emulsion generation increases. In summary, the data we collected and measured for both collagen and alginate emulsions follow the same behavior of deionized water in mineral oil, as was thoroughly discussed in the “Single Emulsion Generation” section.

The double emulsion generation of collagen emulsions in mineral oil in deionized water was conducted using the 3D printed device. Three different inner flow rates of collagen with constant middle and outer flow rates were used to observe the possibility of generating compound biological emulsions using our device. The experiments were conducted at 100 μL/min of middle flow rate and 1000 μL/min of outer flow rate. We could obtain an average inner emulsion size of 800.33 ± 128.6 μm at 10 μL/min and an average outer emulsion size of 2704.25 ± 420 μm at 10 μL/min of collagen flow rate. By increasing the collagen flow rate to 20 and 30 μL/min, the average inner emulsion size increased to 1198 ± 53 μm and 1383.3 ± 12.5 μm, respectively. As we discussed in the “Double and Compound Emulsion Generation” section, the increase in size of the inner emulsion was expected, since the middle flow rate plays the role of a continuous flow rate for the inner dispersed flow rate. The average size of the outer emulsions in these two inner flow rates was 2539 ± 110 μm for the 20 μL/min flow rate and 2717.9 ± 173 μm for the 30 μL/min flow rate.

## 4. Conclusions

In conclusion, we designed and produced a monolithic 3D printed axisymmetric co-flow device for generating single and double/compound emulsions. This device was successfully tested using deionized water and mineral oil as two immiscible fluids, producing single emulsions with an average diameter of 1838 to 3000 μm in different dispersed and continuous flow rates. In addition, double/compound emulsions were produced with outer emulsions having an average diameter of 2150 to 2930 μm and inner emulsions having an average diameter of 488 to 1120 μm by keeping the inner phase flow rate constant and varying the middle and outer flow rates. We also achieved compound emulsions and encapsulate more than one—and up to six—inner emulsions inside the larger emulsion using this device. In addition, we tested generating bio emulsions using our device, successfully producing collagen and alginate emulsions in mineral oil. In addition, we encapsulated double emulsions of alginate in mineral oil in deionized water. Overall, our emulsion generator would help any user to produce emulsions and achieve favorable emulsion size by varying the flow rates and the size of the orifice. Moreover, the device is designed in a “plug-and-play” manner, and it is fabricated using a commercial and low-cost Stereolithography 3D printer. These design and fabrication features offer any user an accessible and easy-to-utilize device to produce single, double, and compound emulsions.

## Figures and Tables

**Table 1:** The compound emulsions generated in different sets of middle and outer flow rates with various numbers of water emulsions encapsulated in mineral oil. The scale bar for each picture is 500 μm.

	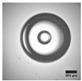	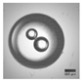	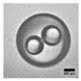	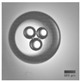	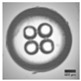	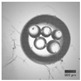	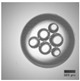
Middle Phase Flow Rate (μL/min)	10	30	10	25	30	20	25
Outer Phase Flow Rate (μL/min)	150	60	60	75	30	150	75
Outer Emulsion Size (Micrometers)	2243	2511	2466	2325	2932	2218	2432
Inner Emulsion Size (Micrometers)	618	519, 684	883, 931	562, 518, 568	636, 683, 649, 675	585, 492, 467, 567, 627	455, 559, 612, 510, 572, 416
Average Inner Emulsion Size (Micrometers)	618	601.5	907	549.33	660.75	547.6	520.67
Standard Deviation (Micrometers)	0	82.5	24	22.29	19.03	59.44	68.10
